# The Effect of Angipars on Diabetic Neuropathy in STZ-Induced Diabetic Male Rats: A Study on Behavioral, Electrophysiological, Sciatic Histological and Ultrastructural Indices

**DOI:** 10.1155/2014/721547

**Published:** 2014-12-29

**Authors:** Nasser Zangiabadi, Hossein Mohtashami, Mahboobeh Hojatipour, Mandana Jafari, Majid Asadi-Shekaari, Mohammad Shabani

**Affiliations:** ^1^Neuroscience Research Center, Institute of Neuropharmacology, Kerman University of Medical Sciences, Tahmassebabad Crossroad, Ebne-Sina Street, Kerman 76198-13159, Iran; ^2^Afzal Research Center, Kerman, Iran

## Abstract

Diabetes mellitus is the most common metabolic disease with a high prevalence rate in human society that eventually leads to the peripheral nervous system complications in a great number of patients. In the present study, the effects of Angipars on nerve conduction velocity, histological alterations, and behavioral indices were investigated. Diabetes was induced in male rats by intraperitoneal injection of streptozotocin (STZ). Six weeks after STZ injection, animals were divided into five groups control, vehicle, and 3 experimental groups. The vehicle group received 1 mL distilled water daily for two weeks and three experimental groups received, respectively, intraperitoneal injection of 5, 10, and 20 mg/kg Angipars daily for two weeks. Intraperitoneal injection of Angipars, in some extent, could significantly improve behavioral indices of the experimental groups as compared to the vehicle group. Furthermore, mean nerve conduction velocity in the vehicle group showed significant difference with that in the control and the 2nd experimental groups; therefore, Angipars could increase nerve conduction velocity in neuropathic rats. Overall, Angipars exerted positive effects on the treatment and reduction of physiologic symptoms and improvement of sciatic morphological injuries in neuropathic rats.

## 1. Introduction

Diabetes mellitus is a chronic metabolic disorder that leads to long-term complications affecting some tissues such as heart, kidney, retina, and peripheral nerves [1, 2]. Chronic hyperglycemia leads to the dysfunction of several organs especially eye, kidney, heart, and vessels. Peripheral neuropathy is one of the common complications of diabetes which in turn increases the risk of other diabetes complications such as foot ulcers and amputation [3, 4]. Almost more than half of diabetic patients suffer from different forms of neuropathy after passing 1-2 decades of their disease [5]. Abnormalities of polyol pathway and defects of protein kinase C metabolism which cause nerve demyelination have also been described in diabetic peripheral neuropathy [6]. Based on these mechanisms of injury, various prevention and treatment strategies have been already suggested and are under investigation. For instance, the effects of several antioxidants such as vitamin E [7], melatonin [8] and date extract [1], fatty acid contained diets like Omega 3 [9], aldose reductase inhibitors [10] and also statins compounds such as atorvastatin [11] have been investigated.

*Melilotus officinalis* has been introduced as a component of a new drug by trade name of Semilil (Angipars). Previous studies have shown beneficial effects of Semilil such as improvement of blood circulation, reduction of inflammation, and improvement of lymphedema and immune system [12, 13]. Results of clinical trials on Semilil indicated its safety and efficacy in human diabetic foot ulcer [14, 15]. This drug has been found to have strong antioxidant components such as 7-hydroxycoumarin, flavonoids, and oleaneneglucuronide [16, 17]. Since some of these properties (improvement of blood circulation, anti-inflmmation, and antioxidation) may be useful in the treatment of the damages inevitably followed by diabetic peripheral neuropathy, in this study the protective effects of Angipars on diabetic neuropathy in male rats were investigated.

## 2. Materials and Methods

### 2.1. Medicines

Streptozotocin was purchased from Sigma Company and Angipars was purchased from Pars Rooz Company (Tehran, Iran). Angipars contains compounds such as 7-hydroxycoumarin and flavonoids and is soluble in distilled water.

### 2.2. Animals and Experimental Protocols

In this study, 40 mature male Wistar rats weighed 250–300 g with the approximate age of 2.5–3 months were used. Animals were prepared from the animal house of Kerman Neuroscience Research Center and kept in the same place. During the study period, standard pellet diet and water were given to experimental animals ad libitum and they were kept in the laboratory animal house under specific pathogen-free (SPF) and constant temperature (25 ± 1°C) conditions and a 12 h light-dark cycle (lights on at 06:30 h). Special care was provided to minimize animal suffering and to reduce the number of animals used to the minimum required for statistical accuracy. Animals were acclimatized to laboratory conditions before the tests. All experiments were carried out 09:00–13:00 h. Diabetes was induced by intraperitoneal injection of 65 mg/kg streptozotocin (prepared in 100 mmol/L sodium citrate buffer, pH = 4.5) [18]. One week after streptozotocin injection, blood sugar was measured by Easygluco instrument. For this, animal's tail was scratched and one drop of its blood was poured on the instrument strip. Then, the amount of blood sugar was read on the monitor. Six weeks after ensuring of diabetes induction (FBS > 200 mg/dL), animals were divided into the five groups:

1. control group: nondiabetic rats that used standard food and water during the experiments and received no medicine;
2. vehicle group: streptozotocin-induced diabetic rats that received 1 mL distilled water daily for two weeks;
3. the first experimental group: streptozotocin-induced diabetic rats that received 5 mg/kg Angipars daily for two weeks (dissolved in distilled water);
4. the second experimental group: streptozotocin-induced diabetic rats that received 10 mg/kg Angipars daily for two weeks;
5. the third experimental group: streptozotocin-induced diabetic rats that received 20 mg/kg Angipars daily for two week (Six weeks after diabetes induction, Angipars and distilled water injections were started).

### 2.3. Physiological Investigations

Tail flick test is one of the standard tests for measuring the rate of nociception. In this test, thermal light with the intensity of 5 is directed on the distal part of animal's tail by Tail flick instrument (LE7406) and the time from the onset of stimulation to sudden tail's withdrawal (tail flick latency) is measured. In order to avoid tissue damage, the stimulus cut-off was set to the maximum of 10 seconds. Mean of three measures is reported as tail flick latency. The interval of three measurements was 5 minutes [19, 20]. Open field test was used in order to investigate the effect of diabetic neuropathy on explorative behavior of diabetic rats and the probable protective effect of Angipars. Eight weeks after experiments onset, animals' explorative behavior in a 90∗90∗40 cm box was investigated by camera (TSE). At the end of 8th week after diabetes induction, animals were put in the center of defined area and for 5 minutes their explorative behavior including the distances paved in the horizontal direction in the center and peripheral areas (Total Distance Moved = TDM) and the duration of staying in the center and peripheral areas as well as the frequency of grooming and rearing were investigated [21, 22].

### 2.4. Nerve Conduction Velocity Measurements

Eight weeks after diabetes induction, animals were anesthetized by the injection of 50/20 mg/kg ketamine/xylazine solution. Then, their legs were shaved. During this study, rectal temperature was kept at 37°C to decrease the animal's stress due to anesthesia.

In a temperature controlled environment (25 ± 1°C), small incisions were made in the right sciatic notch and ankle. The nerve conduction velocity of sciatic-tibial motor nerve was measured through proximal stimulations close to sciatic notch and distal stimulations close to knee by bipolar needle electrodes (PowerLab/ML 856, AD instrument, Sydney, Australia, frequency: 20 Hz, duration: 0.1 ms, amplitude: 1.5 V). After the first stimulation, evoked potential of plantar hind foot muscles was recorded by monopolar electrodes. This value was the primary biphasic response in M wave which is a direct motor response based on stimulation of motor fibers. MNCV was calculated by dividing the distance between the two stimulated points (mm) by the time difference of two stimulated points (m/s) [1, 20].

### 2.5. Histological Investigations (Histopathology)

After the previous experiments, animals were deeply anesthetized by 400 mg/kg chloral hydrate and after cardiac perfusion with normal saline and Buen fixative, and 1 cm of sciatic nerve was dissected and put in Buen fixative. After 48 hours, samples underwent tissue processing and were paraffin-embedded. Then, 4 *μ*m slices were prepared and stained with hematoxylin-eosin and investigated under light microscope (∗400) [23]. In this investigation, the axon of neurons in the sciatic horizontal section was studied for edema, demyelination, and remyelination [24].

### 2.6. Morphological Study of Sciatic Nerve

In order to study morphological alterations resulting from diabetic neuropathy, sciatic nerve was isolated and divided into two 1 mm sections. The sections were immediately fixed in 2.5% glutaraldehyde solution in 0.1 m phosphate buffer solution for 24 hours. Samples were washed with the mentioned solution three times and put in 1% osmium tetra oxide for 2 hours and after dehydration were embedded in Epon812 resin using acetone. Semithin sections with 300 nm thickness and thin sections with 70 nm thickness were prepared, stained, and studied under electronic microscope [1].

### 2.7. Statistical Analysis

Parametric paired *t*-test was used for comparison of coupled primary and secondary variables and in order to compare quantitative variables among groups ANOVA was applied. Tukey test was used in the case of significant difference and in the case of rejection of null hypothesis, nonparametric Kruskal-Wallis test was applied.

## 3. Results

### 3.1. Metabolic Parameters

All diabetic rats showed high blood sugar and weight gain disorder in the 8th week after STZ injection. As it has been presented in Table 1, weight of all diabetic animals compared to the control group had significantly decreased in the 8th week (Table 1). Treatment of diabetic neuropathy with Angipars cannot significantly affect blood sugar level and weight gain of diabetic rats in comparison to the diabetic and vehicle groups (Table 1).

### 3.2. The Effects of Angipars on Tail Flick Test

**Figure 1:**
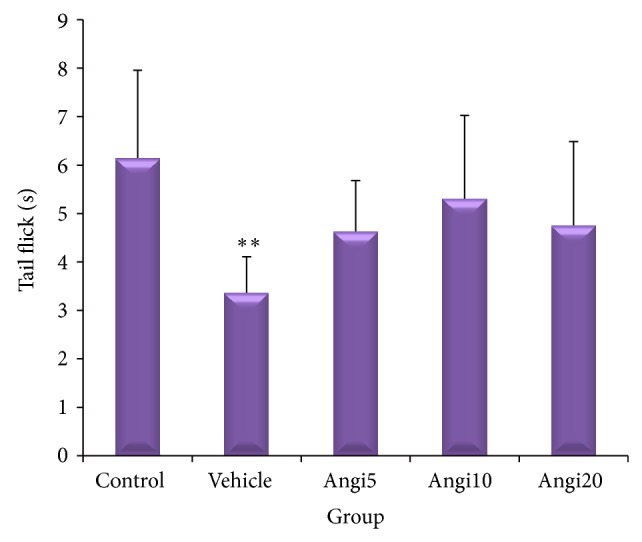
Effect of Angipars (5, 10, and 20 mg kg day^−1^, ip, for 2 weeks) on the pain threshold values in streptozotocin-injected diabetic rats subjected to tail flick. ^**^ *P* < 0.01 as compared to control group. There are no significance effects between Angipars-treated groups and vehicle group. Values are expressed as mean ± SEM (*n* = 8 rats in each group).

Diabetic neuropathy caused significant decrease (*P* < 0.01) in reaction to pain and tail flick latency time in vehicle group compared to the control group, but all three Angipars-treated groups showed no significant difference with control and vehicle groups in this regard (Figure 1).

### 3.3. The Effects of Angipars on Open Field Test

**Figure 2:**
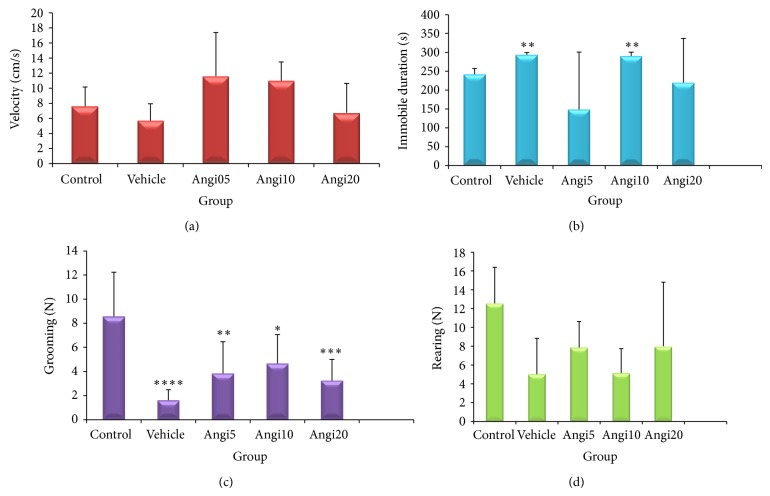
Effect of Angipars (5, 10, and 20 mg kg day^−1^, ip, for 2 weeks) on explorative behavior of rats in open field test. (a) Velocity, (b) immobile duration, (c) grooming, and (d) rearing. ^*^ *P* < 0.05, ^**^ *P* < 0.01, ^***^ *P* < 0.001, and ^****^ *P* < 0.0001 as compared to control group. Data are the mean ± SEM.

The analysis of data related to “velocity” and “rearing” in open field test showed no significant difference among studied groups (Figure 2). In regard to mean immobile duration, vehicle and experimental group that received 10 mg/kg Angipars showed significant difference with the control group, while there are no significance effects on Angipars-treatment groups and vehicle group.

In regard to mean grooming, vehicle and all experimental groups that treatment with Angipars showed signifiant diffrence with the control group, while there are no signifiance differences among Angipars-treatment groups and vehicle group.

### 3.4. The Effects of Angipars on Nerve Conduction Velocity Alterations

**Figure 3:**
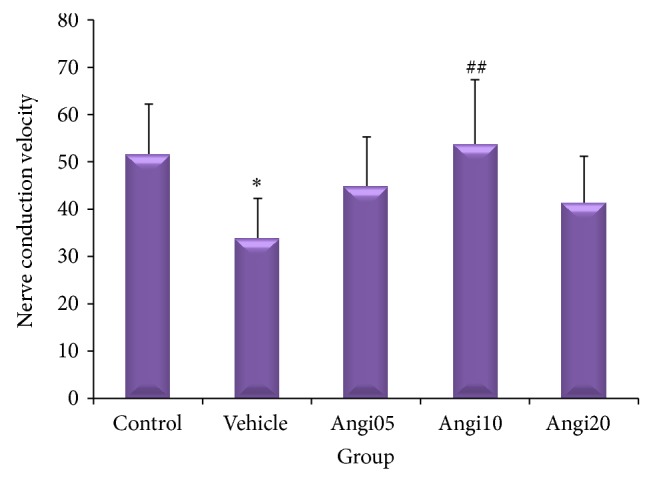
Effect of Angipars (5, 10, and 20 mg kg day^−1^, ip, for 2 weeks) on motor nerve conductive velocity (NCV) in rats. ^*^ *P* < 0.05 as compared to control group; ^##^ *P* < 0.01 as compared to vehicle group. Data are the mean ± SEM.

Mean nerve conduction velocity of vehicle group showed significant difference with those of control group and the experimental group that received 10 mg/kg Angipars. The vehicle group compared to the control group had lower mean nerve conduction velocity. Significant increase of mean nerve conduction velocity in the experimental group compared to the vehicle group shows that Angipars has significant effect on improving nerve conduction velocity in neuropathy-induced male rats (Figure 3). It should be also mentioned that, according to Figure 3, Angipars at doze of 10 mg/kg is more effective than dozes of 5 and 20 mg/kg in improving nerve conduction velocity.

### 3.5. The Effect of Angipars on Morphological Alterations of Nerve Myelin

**Figure 4:**
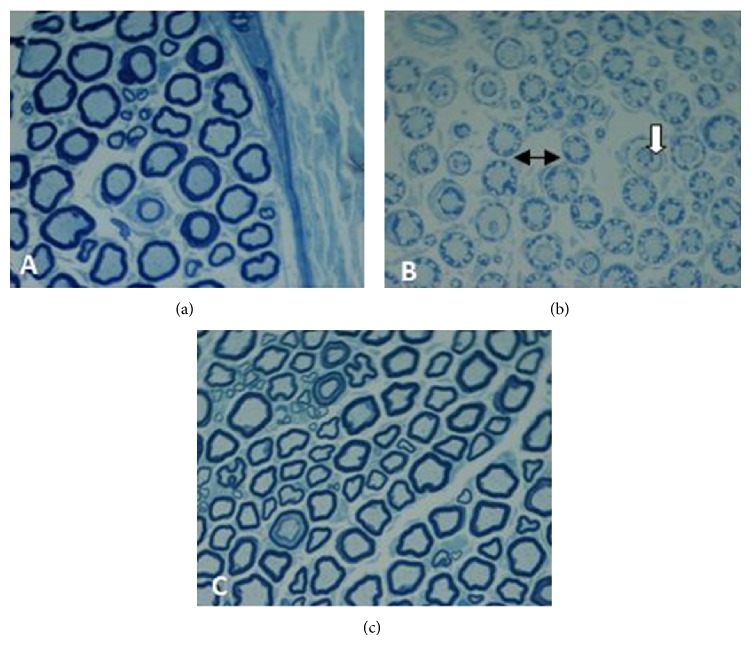
Light micrograph of transverse semithin sections of rat sciatic nerves. (a) Control group: myelinated nerve fibers are in normal structure and morphology. (b) Diabetic group: nerves fibers show some abnormalities such as myelin splitting (white arrow) and swelling (two-sided arrow). (c) The Angipars-treated group (10 mg kg day^−1^, ip, for 2 weeks). In Angipars-treated group, the proportion of nerve fibers with abnormalities was reduced. Magnification ×400.

Light microscopic investigations showed normal structure and morphology of myelin in the control group, but, in the vehicle group, edema and myelin sheath splitting were observed. Increase in the number of fibers with abnormal myelin was observed in the vehicle group. Treatment with Angipars could prevent abnormal cases in a wide extent. Mean nerve fiber diameter in the control and vehicle groups showed significant difference with three Angipars-treated groups. Mean axon diameter in the vehicle group significantly decreased compared to control group while treatment with Angipars could stop axon diameter changes. Angipars 5-received groups had also significant difference with the vehicle group in regard to the mean axon diameter. Mean myelin sheath diameter in the control group showed no significant difference in comparison to the other groups (Table 2) (Figure 4).

**Figure 5:**
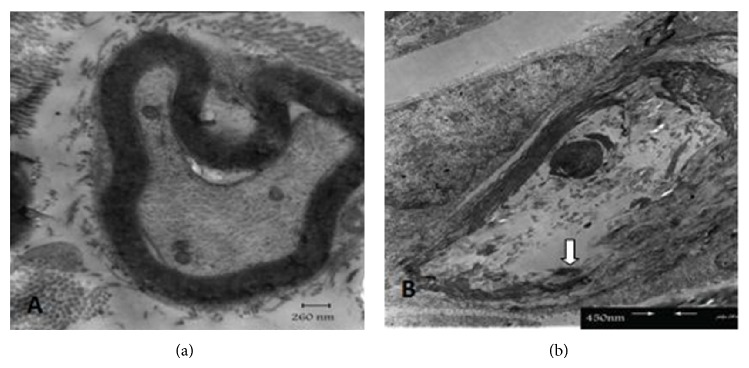
Electron micrograph of rat sciatic nerves. (a) Control group: myelinated fiber with normal structure and morphology. Axon is in normal condition too. (b) Diabetic group: myelinated fiber shows myelin splitting (white arrow) and axoplasm disintegrity.

Ultrastructural evaluation of sciatic nerves with electron microscopy confirmed the light microscopy findings. In addition, some evidences of axonal degeneration such as mitochondrial swelling and disintegration of neurofilaments were observed under transmission electron microscope but degeneration, reaction, and proliferation of Schwann cells were not observed in any groups (Figure 5).

## 4. Discussion

In the present study, the effects of Angipars at dozes of 5, 10, and 20 mL/kg on diabetic neuropathy in streptozotocin-induced diabetic male rats were investigated through behavioral and electrophysiological indices and sciatic histopathology. According to the obtained results, injection of 10 mL/kg Angipars for two weeks has the most positive effects on the treatment and decrease of physiological symptoms of diabetic neuropathy in male rats. Although intraperitoneal injection of Angipars for two weeks could not cause significant changes in some behavioral variables, as compared to the vehicle group improvement of these variables was considerable. These positive findings, in association with significant effect on tail flick test result, can show the efficacy of Angipars on behavioral signs of neuropathy in diabetic rats.

In Shimada et al. study, NCV and F-wave latency have been reported as the best criteria for studying diabetic neuropathy in clinical trials [25]. NCV reduction is an index of neuropathy in experimental diabetes and peripheral neuropathy is attributed to more than 15% decrease of NCV in myelinated sensory or motor nerves [26]. In the present study, mean NCV showed 65% decrease in the vehicle group as compared to the control group. This shows high rate of neuropathy in diabetic animals. It was observed that intraperitoneal injection of Angipars for two weeks has significant effect on NCV improvement in diabetic male rats [1]. The loss of significant protection from diabetic neuropathy in MNCV with the highest doses of Angipars suggested the possibility that Angipars receptor subtypes may have different functional and/or opposing effects.

The presence of abnormal fibers in sciatic nerve with axonal degeneration and myelin breakdown is one of the symptoms of STZ-induced diabetic rats [27]. In fact, one of the major causes of reduction of nerve activity in the process of diabetic neuropathy is morphological alterations which appeared due to the metabolic disorders of the nerve. Zangiabadi et al. in their study on the effect of date extract on abnormal fibers of sciatic nerve undergoing axonal degeneration and myelin breakdown in STZ-induced diabetic mice concluded that these pathological alterations basically cause axonal degeneration in sciatic nerve after STZ injection. They reported significant decrease of MMFD and also MSD worsening in sciatic nerve of STZ-induced diabetic mice that could be improved by date extract [1]. In the present study, too, morphological changes of sciatic nerve such as axonal degeneration and myelin splitting were studied in diabetic rats after 8 weeks of STZ injection. Morphological observations showed remyelination after Angipars injection. Data related to axon diameter showed significant increase of this index in the vehicle group compared to the Angipars-treated groups, while this index showed significant decrease in the vehicle group in comparison to the control group. This finding shows the efficacy of Angipars in the improvement of axonal degeneration of nerve fibers in STZ-induced diabetic rats. Moreover, investigations on MSD showed that treatment of diabetic neuropathy with Angipars injection helps remyelination in diabetic rats. In the present study, decrease of myelin diameter in the vehicle group compared to the Angipars-treated groups shows high rate of remyelination in the neuropathy process in these groups. In whole, increase of myelin and axon diameter in the Angipars-treated groups has caused increase of mean myelin fiber diameter (MMFD) and improvement of the nerve. One of the major mechanisms in the physiopathology of neuropathy is a biochemical pathway named polyolpathway. Since neurons and capillaries' membranes are not insulin dependent in transporting glucose, great amount of glucose enters cells in diabetes. In neurons, glucose is converted to sorbitol by aldose reductase enzyme and sorbitol accumulation increases the production of free radicals such as hydroxyl-hydrogen peroxide-superoxide that eventually leads to cell damage. Based on this mechanism of injury, different preventive and treatment approaches are suggested and are under research. One of them is the role of antioxidants in preventing neuropathy [28, 29]. Angipars is one of the medicines that its antioxidant properties have been proposed. Antioxidant effects of coumarin and flavonoids compounds have been investigated and their efficacy has been proved in experimental studies. Larijani et al. in their study on antioxidant effects of Angipars have concluded that this medicine contains substances such as hydroxycoumarin and flavonoids [30]. Angipars contains an herbal compound with special effects on diabetic foot ulcer. According to the manufacturers, some of its compounds are urea, selenium, fructose, and* Melilotus officinalis*. Asadi-Shekaari et al. have reported that urea can improve cerebral oxygenation and act like mannitol that is well known as a neuroprotective agent. Selenium is a famous antioxidant agent that can have neuroprotective activity.* Melilotus officinalis* can decrease the activity of circulating phagocytes and has anti-inflammatory, antiedematous, and antioxidant activities [31]. The majority of recent investigations have shown that one of the most common problems in diabetic patients is delayed wound repair due to vascular insufficiency and decreased blood flow [32, 33]. Establishing appropriate blood flow over the affiliated limb is the robust goal of the treatment that eminently improves healing process [34, 35]. Some investigations for pathophysiological effect of Angipars have revealed that this drug probably improves total tissue blood flow and oxygenation. Decreased blood flow may result in insufficient oxygen delivery, which makes healing impossible unless angioplasty or a vascular bypass is performed. Another contributing factor is microvascular disease. It is generally considered that improved blood supply, through angiogenesis, for example, may improve ulcer healing. Furthermore, Larijani et al. have concluded that Angipars increases the total tissue blood flow and consequently increases oxygenation due to the new angiogenesis in the tissue [30]. In diabetic neuropathy, blood flow decrease and oxygen shortage result in anaerobic metabolism, energy loss, ATP decrease, and accumulation of hypoxanthine in ischemic cells. Lack of energy affects ATPase ion pump of cell membrane and causes sodium, calcium, and water accumulation and consequently cell edema [36]. Therefore, angiogenesis is one of the other mechanisms that can be suggested for explaining the efficacy of Angipars in the treatment of diabetic neuropathy.

## 5. Conclusion

According to the obtained results in the present study and the results of previous studies, injection of Angipars for two weeks, in spite of not being effective on some indices, has some positive effects on the treatment and decrease of physiologic symptoms of neuropathy in male rats. Moreover, it should be mentioned that Angipars at doze of 10 mL/kg has the most positive effects on some indices of diabetic neuropathy.

## Figures and Tables

**Table 1:** Body weight and blood glucose levels of all groups.

Animal group	Body weight (g)		Blood glucose (mg/dL)	
	Before STZ injection	End experiment	Before drug injection	End experiment
Control (8)	270 ± 13.9	276.25 ± 13.29	133 ± 33.41	132.87 ± 35.62
Vehicle (8)	260 ± 21.60	237.14 ± 24.97^*^	423.71 ± 108.85	491.42 ± 87.83
Angipars 5 (8)	257.1 ± 21.38	210 ± 45.82^*^	457.1 ± 115.71	492.85 ± 137.44
Angipars 10 (8)	275.5 ± 14.24	272.22 ± 22.79	391.33 ± 66.64	459.22 ± 94.98
Angipars 20 (8)	266.25 ± 14.07	251.87 ± 17.30	359.87 ± 65.21	471.25 ± 58.66

^*^Data are the mean ± SEM (*n* = 8).

**Table 2:** The effect of Angipars on histomorphometric parameters of rat sciatic nerve.

Groups	*N*	MMFD (*μ*m)	AD (*μ*m)	MSD (*μ*m)
Control	6	6.13 ± 0.88	6.91 ± 1.45	5.35 ± 0.34
Vehicle	6	9.45 ± 0.45^****^	4.59 ± 0.76^**^	4.98 ± 0.71
Angipars 5	6	12.59 ± 1.05^∗∗∗∗#^	6.84 ± 0.99^##^	5.65 ± 0.92
Angipars 10	6	11.65 ± 1.22^∗∗∗∗#^	5.75 ± 1.20	5.91 ± 0.82
Angipars 20	6	11.81 ± 0.73^∗∗∗∗#^	6.00 ± 0.77	5.83 ± 0.63

*N*: number of animals; MMFD: mean-myelinated fiber diameter; AD: axon diameter, MSD: myelin sheath diameter. Data are presented as mean ± SEM. ^*^ *P* < 0.05, ^**^ *P* < 0.01, and ^****^ *P* < 0.0001 as compared to control group. ^##^ *P* < 0.01, ^###^ *P* < 0.001, and ^####^ *P* < 0.0001, as compared to vehicle group.
